# Nuclear Targeted Peptide Combined With Gambogic Acid for Synergistic Treatment of Breast Cancer

**DOI:** 10.3389/fchem.2021.821426

**Published:** 2022-01-28

**Authors:** Wenli Dang, Pan Guo, Xunan Song, Ying Zhang, Nan Li, Changxiang Yu, Bin Xing, Rui Liu, Xintao Jia, Qingqing Zhang, Xiaojiao Feng, Zhidong Liu

**Affiliations:** ^1^ Tianjin State Key Laboratory of Component-based Chinese Medicine, Tianjin University of Traditional Chinese Medicine, Tianjin, China; ^2^ Heihe Laboratory of Modern Chinese Medicine, Tianjin University of Traditional Chinese Medicine, Tianjin, China; ^3^ Engineering Research Center of Modern Chinese Medicine Discovery and Preparation Technique, Ministry of Education, Tianjin University of Traditional Chinese Medicine, Tianjin, China

**Keywords:** anti-tumor, breast cancer, gambogic acid, liposome, nuclear targeted peptide (CB5005N)

## Abstract

As a natural compound, gambogic acid (GA) emerged a shining multi-target antitumor activity in a variety of tumors. Whereas its poor solubility and non-specific effect to tumor blocked the clinical application of this drug. Herein, we reported a simple and effective strategy to construct liposome modified with nuclear targeted peptide CB5005N (VQRKRQKLMPC) *via* polyethylene glycol (PEG) linker to decrease the inherent limitations of GA and promote its anti-tumor activity. In this study, liposomes were prepared by thin film hydration method. The characterization of formulations contained particle size, Zeta potential, morphology and encapsulation efficiency. Further, *in vitro* cytotoxicity and uptake tests were investigated by 4T1 and MDA-MB-231 cells, and nuclear targeting capability was performed on MDA-MB-231 cells. In addition, the *in vivo* antitumor effect and biological distribution of formulations were tested in BALB/c female mice. The GA-loaded liposome modified by CB5005N showed small size, good uniformity, better targeting, higher anti-tumor efficiency, better tumor inhibition rate and lower toxicity to normal tissues than other groups. *In vitro* and *in vivo* research proved that CB5005N-GA-liposome exhibited excellent anti-tumor activity and significantly reduced toxicities. As a result, CB5005N-GA-liposome nano drug delivery system enhanced the tumor targeting and antitumor effects of GA, which provided a basis for its clinical application.

**GRAPHICAL ABSTRACT:**
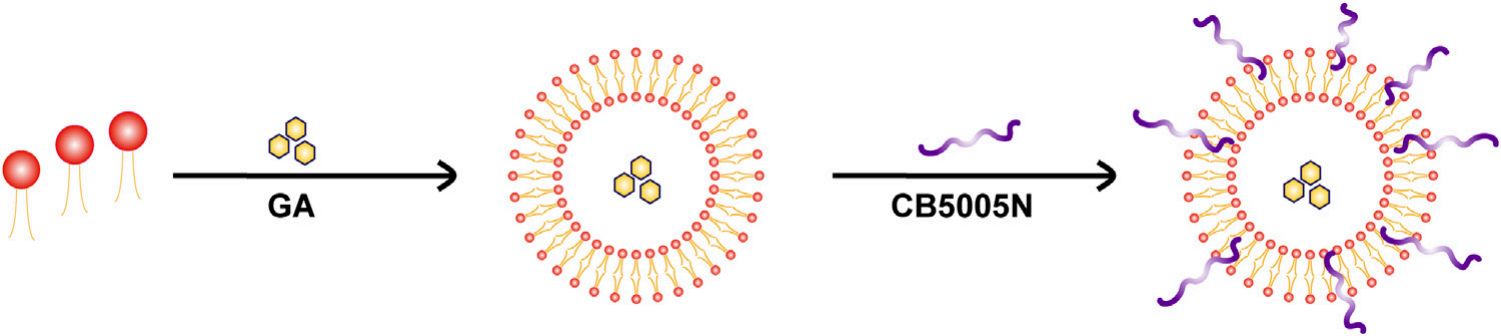
The schematic diagram of CB5005N-GA-liposome.

## 1 Introduction

Breast cancer is the second leading reason of cancer death closely followed lung cancer in females and males (Bray et al., 2018; Gu and Li, 2020; Juan et al., 2020). Recently, the multi-target effects of traditional Chinese medicine compounds in treatment of cancer have received widespread attention, providing new ideas for cancer prevention and treatment (Wang et al., 2014).

At the moment, it has been found that a range of monomers of traditional Chinese medicine with anti-tumor activity, such as gambogic acid, neogambogic acid, curcumin, matrine and so on (Cheng et al., 2020; Hatami et al., 2020; Zha et al., 2020; D'Angelo et al., 2021), compared with single-target chemical drugs, the anti-tumor active components of traditional Chinese medicine provide a new idea for tumor prevention and treatment through the synergistic regulation of multi-targets and multi-pathways.

Gamboge, as a pigment used in ancient Chinese drawings, also used in folk medicine (Wang and Chen, 2012). GA, the most abundant ingredient of Gamboge, is a xanthone structure extracted from the dry brownish gamboge resin secreted from the *Garcinia hanburyi* tree in Southeast Asia, and has inherent anti-cancer properties, which exhibits remarkable programmed cell death, cell cycle arrest, anti-angiogenesis and anti-inflammatory effects (Kashyap et al., 2016; Pandey et al., 2016). Because of these reasons, the anti-tumor effect had particularly attracted widespread attention, GA could inhibit various cancer cell’s growth, including colorectal cancer, breast cancer, liver cancer, lung cancer, etc. (Li et al., 2010; Yan et al., 2012; Zhou et al., 2013; Xu et al., 2018; Zhao et al., 2020), which might be related with modulating the signaling pathways of protein kinase B (AKT)/mammalian target of rapamycin (mTOR), c-Jun N-terminal cvvkinase-1 (JNK-1), nuclear factor kappa-B (NF-κ B), AKT/forkhead box protein O1(FOXO1)/BIM and so on. And some studies had shown that GA has low toxicity to normal non-tumor cells, but it can effectively kill tumor cells and selectively induce cancer cell apoptosis of tumor cells (Yang et al., 2007). Although GA has been approved for phase II clinical trials by the China Food and Drug Administration (CFDA) as an anti-cancer agent for lung cancer and other solid tumor therapy (Chi et al., 2013), the poor water solubility, strong irritation, short half-life and low bioavailability are the “Achilles’ heel” for its clinical application. The new drug delivery system based on nanotechnology could usefully encapsulate GA to overcome the poor water solubility and enhance the therapeutic effects in cancer therapy.

The application of liposome delivery system in tumor therapy has been widely studied. Liposomes are a vesicle structure composed of hydrophilic core and lipophilic phospholipid bilayer, which has good biocompatibility and low toxicity to the host. It can safely and effectively deliver different therapeutic drugs to the tumor site, and can further promote its anti-tumor activity (Mapossa et al., 2021; Mirzavi et al., 2021). In recent years, targeting-peptide modified liposomes had been widely studied to achieve active targeting of drugs through surface-attached targeting-peptide, which assist in the targeted delivery of drugs to tumor cells or tumor-related stromal cells, and in order to enhance the selectivity of drug delivery and the accumulation of drugs in the tumor site (Yan et al., 2020).

With increasing research about diseases at the histological, cytological to organelle level, targeting organelle therapy has gradually been envisioned as an approach to overcome the shortcomings of poor specificity and multiple toxic side effects on tissues and cell-level treatments using the currently available therapy. The peptide CB5005N(VQRKRQKLMPC) is designed according to the functionalized NF-κ B peptide inhibitor SN50 (Lin and Budu, 2008). The cell penetration peptide CB5005 is divided into two sequences, one is the membrane penetration sequence CB5005M (sequence KLKLALALALA), which can penetrate the cell membrane, and the other is the nuclear localization sequence CB5005N (sequence VQRKRQKLMPC), which does not have the function of penetrating the cell membrane, but can locate the nucleus and prevent the activated NF-κ B protein from entering the nucleus (Zhang et al., 2016). In this experiment, we truncated the nuclear localization sequence CB5005N from the cell penetrating peptide CB5005, and it was supposed to play the role of nuclear localization, so that it could enter into the nucleus while targeted delivery of gambogic acid to the tumor site, so as to further exert its anti-tumor effect.

Based on the above mechanism, the insoluble anti-tumor component GA was encapsulated in the liposome, and the tumor-targeting nanoplatform (CB5005N-GA-liposome) was modified with CB5005N. CB5005N-GA-liposome system had a significant effect on raising the bioavailability, targeted tumor and anti-tumor efficacy of GA were comprehensively evaluated both *in vivo* and *in vitro*.

## 2 Materials and Methods

### 2.1 Materials

GA with purity over 98% was obtained from Chengdu Herb purify Co. Ltd. (Chengdu, China); 1,2-distearoyl-sn-glycero-3-phosphoethanolamine-N-[(polyethylene glycol)-2000] (DPSE-PEG2000), cholesterol and Hydrogenated soybean phosphatidylcholine (HSPC) were derived from AVT (Shanghai, China); anhydrous ethanol (Analytical Pure, Tianjin Concord Technology Co., Ltd.); DSPE-PEG2K-CB5005N peptide was synthesized by China peptide Biotech Co. Ltd. (Shanghai, China); DMSO, Coumarin-6 and Hoechst 33342 from Sigma-Aldrich (MO, United States). 1,10-Dioctadecyl-3,3,3,3-tetramethyl indotricarbocyanine iodide (Dir) was purchased from Biotium Inc. (Hayward, CA); Cell culture plate were purchased from Coring (United States); Cell Counting Kit-8 (CCK-8) was purchased from Dojindo Laboratories (Kumamoto, Japan); Fetal bovine serum (FBS) was purchased from Biological industries (Israel); 0.25% trypsin +0.02% EDTA, Phosphate Buffered Saline (PBS), RMPI-1640 medium, Dulbecco’s modified Eagle’s medium (DMEM) and penicillin-streptomycin were obtained from Gibco (Invitrogen, United States).

### 2.2 Cell Culture

Human breast carcinoma MDA-MB-231 cells and mice breast cancer 4T1 cells were purchased from American Type Culture Collection (ATCC). MDA-MB-231 cells were cultured in RPMI-1640 medium containing 15% FBS, and 4T1 cells were cultivated in DMEM medium added to 10% FBS, these cells were cultivated in a humidified atmosphere containing 5% CO_2_ at 37°C. All cell lines were detected without *mycoplasma* contamination.

### 2.3 Animals

BALB/c nude mice (female, 5–8 weeks, 17–19 g) were supplied by Beijing Weitonglihua Laboratory Animal Technology Co., Ltd, License Number: SCXK (Beijing) 2016–0006); and BALB/c mice (female, 4–6 weeks, 16–20 g) were purchased from Beijing HFK Bioscience Co., Ltd. (Beijing, China). Fed at a controlled temperature of 25 ± 1°C and a relative humidity of 45–55% for a week to adapt to the new environment. All animal experiments were performed in accordance with guidelines evaluated and approved by the Ethics Committee of Tianjin University of Traditional Chinese Medicine (Document number: TCM-LAEC2021145).

### 2.4 Preparation of CB5005N-GA-Liposome

Used the thin film hydration method to prepare liposomes. To get the liposomes modified with CB5005N, HSPC, cholesterol and CB5005N were carefully weighed according to the ratio of exposure to quality 3:1:0.5, respectively. Those were mixed to the oil phase, then transferred to the eggplant-shaped bottle under the condition of 60°C water bath, in order to evaporate the organic solvent to make the lipid film uniformly. Then added deionized water and shook, hydrated in water bath at 60°C for 1 h, until the lipid membrane fell off completely. Under the ice bath, the solution was crushed for 8min with ultrasonic cell crusher (working for 5 s, intermittent 3 s for 60 cycles, 100 W), the liposomes, as a yellowish homogeneous solution with milky light, was obtained. When prepared GA-liposomes, Dir-liposomes or Cou-6-liposomes, GA, Dir and Cou-6 were mixed in the oil phase.

### 2.5 Characterization of Liposomes

#### 2.5.1 Particle Size, Zeta Potential and Morphology

Size, polydispersity index (PDI) and zeta potential measurements of liposomes including Blank-liposome, GA-liposome and CB5005N-GA-liposome were measured at room temperature through a dynamic light scattering (DLS) technique using Nano ZS (Malvern Instruments, UK). The morphology was observed under transmission electron microscope (TEM) (JEM-1200EX (120 KV), JEOL, Japan) with negative stain method.

#### 2.5.2 Determination of Encapsulation Efficiency

Determination of encapsulation efficiency (EE) of GA by ultrafiltration centrifugation and high-performance liquid chromatography (HPLC) methods. To measure the total concentration of GA, liposomes were dissolved in acetonitrile containing 0.2% NaCl and sonicated for 40 min, and the concentration of free GA was determined by the centrifugation of liposomes at 5,000 revolutions per minute (rpm) for 20 min. Then the encapsulation efficiency was determined *via* HPLC (LC-20A, Shimadzu, Japan) on a reverse phase C_18_ column (250 mm × 4.6 mm, 5 μm) at 30°C. The mobile phase contained (A) acetonitrile (B) water including 0.1% formic acid (A: B = 9: 1). The injected volume was 10 μL and the flow rate was 1.0 ml/min. Verified the specificity, precision, linearity, repeatability and stability of the method. The EE was calculated using the following equation:

EE (%)=( 1 − W freeWtotal )× 100%

Where *W*_*total*_ and *W*_*free*_ were the total weight of GA in the liposome and the weight of free GA, respectively.

#### 2.5.3 Thermal Analysis Studies

Thermal analysis of GA, Blank liposome, their physical mixture, GA-liposome and CB5005N-GA-liposome was detected by a differential scanning calorimetry (DSC, PerkinElmer Inc. United States). The samples were heated to a temperature of 300 °C under nitrogen atmosphere with a constant heating rate of 10°C/min.

#### 2.5.4 In vitro Hemolysis Test and Its Stability

The safety of liposomes was evaluated by *in vitro* hemolysis test. Used the centrifuge tube containing anticoagulants, 10–20 ml fresh blood was taken from healthy rabbits, and the blood was stirred with glass rods to remove fibrinogen. Added 10 times of normal saline solution, shook well and centrifuged at 1,500 rpm for 20 min under the condition of 4°C. Then discarded the upper normal saline and washed twice with normal saline until the upper solution was yellowish or colorless. The obtained red blood cells were prepared into 2% suspension with normal saline for the experiment.

The clean test tubes were arranged on the test tube rack, and the positive and negative controls were deionized water and normal saline, respectively. According to the proportion of Table 1, 2% red blood cell suspension, deionized water, normal saline, blank liposome, GA-liposome, CB5005N-liposome and CB5005N-GA-liposome were added in turn, shook well and immediately incubated in a constant temperature water bath of 37 ± 0.5°C. Then the supernatants were obtained at 1, 2, 3 and 24 h with 1,500 rpm centrifugation for 10 min. The absorbance of each group was determined under 540 nm condition (Li et al., 2018), and the degree of hemolysis was calculated according to the equation.

$$\mathrm{Hemolysis}\left(\%\right)=\left(\frac{A_{1}-A_{2}}{A_{3}-A_{2}}\right)\times 100\%$$

**TABLE 1:** Blood safety test plus sample table.

Number	2% red blood cell suspension (ml)	Deionized water (ml)	Normal saline (ml)	Blank-liposome (ml)	GA-liposome (ml)	CB5005N-liposome (ml)	CB5005N-GA-liposome (ml)
1	2.5	2.5	—	—	—	—	—
2	2.5	—	2.5	—	—	—	—
3	2.5	—	2.2	0.3	—	—	—
4	2.5	—	2.2	—	0.3	—	—
5	2.5	—	2.2	—	—	0.3	
6	2.5	—	2.2	—	—		0.3

In the above equation, *A*_*1*_, *A*_*2*_ and *A*_*3*_ were the absorbance of the various samples, normal saline and deionized water, respectively.

### 2.6 Cell Uptake

The cell uptake behavior of the liposome formulations was tested on 4T1 and MDA-MB-231, and Coumarin (Cou-6) was selected as a fluorescent probe to be loaded in liposome. The 4T1 and MDA-MB-231 cells were harvested at the logarithmic growth phase and seeded on 96 well plates at a density of 1.2×10^4^ cells/well and 1.0×10^4^ cells/well, followed by incubation for 24 h. Then added free Cou-6-Sol, Cou-6-liposome and CB5005N-Cou-6-liposome at their non-toxic concentration for 4 h to the cells. After washing with cold PBS, per well was added 50 μL Hoechst 33342 (1 μg/ml) to stain the nuclei. Subsequently, cellular internalization analysis of Cou-6 was performed by InCell Analyzer 2500 High connotation Cell Imaging Analysis system (GE Healthcare, United States).

### 2.7 Nuclear Targeting

With a confocal laser scanning microscope (CLSM) observed the Cou-6-Sol, the Cou-6-liposome, CB5005N-Cou-6-liposome located in the nucleus of the MDA-MB-231 cells. The cells (8.0×10^4^ cells/well) were seeded in 12 well plate, followed by incubation for 24 h. Then cells were incubated with a RPMI-1640 medium containing 15% FBS and different formulations for 4 and 8 h. Then washed with cold PBS two times and stained with Hoechst 33342 for 30 min. After the stained cells were washed twice with PBS and observed by CLSM at excitation wavelengths of 346 and 360 nm, and emission wavelengths of 460 and 477 nm for Hoechst 33342 and Cou-6, respectively.

### 2.8 *In vitro* Evaluation of Cytotoxicity

The cytotoxicity of different formulations on 4T1 and MDA-MB-231 cells were assessed by CCK-8 Kit. The cells were seeded at a density of 5.0×10^3^ cells/well onto 96 well plates and incubated overnight at 37°C. The different preparations with final GA’s concentration of 0.1–0.6 μg/ml (4T1) and 0.2–1.0 μg/ml (MDA-MB-231) were added to each well. 24 h later, removed culture medium and added 100 μL/well 10% CCK-8 solution for incubating another 30 min, and measured at wavelength of 450 nm with Spark multifunctional enzyme labeling instrument (TECAN, Austria). Cell viability was calculated as:

$$\mathrm{Cell viability}\left(\%\right)=\left(\frac{A_{s}-A_{b}}{A_{c}-A_{b}}\right)\times 100\%$$

Where, the *A*_*s*_, *A*_*c*_ and *A*_*b*_ respectively referred to the absorbance of the experimental groups, the control group and the blank group.

### 2.9 In-vivo Imaging

The female nude mice (5–8 weeks, 17–19 g) were subcutaneously inoculated with 2.0×10^7^ 4T1 cells for constructing the orthotopic tumor-bearing mice model (Kebebe et al., 2019). Dir, as the fluorescent probe, was loaded in the liposome to assess its biological distribution and tumor targeting *in vivo*. After 10 days of implantation, nude mice were randomly divided into three groups with three mice in each group, while the tumor volume ranging from 200 mm^3^ using the formula (length × width^2^)/2. Then injected different formulations through the tail vein, Dir dose of 0.5 mg/kg. The mice were anesthetized at 1, 4, 8 and 24 h after injection and placed in an *In-Vivo* Imaging System (IVIS; CRi Maestro™ two Maestro™ EX-RRO, American) with wavelength Ex = 748 nm and Em = 780 nm, to observe the fluorescence intensity at the tumor site. The animals were sacrificed after 24 h of imaging.

### 2.10 *In-vivo* Antitumor Efficacy

We inoculated animals in the mammary fat pad with 4T1 cells. Treatments began at day 8, the tumor bearing-mice were randomly divided into five groups (4 mice in each group). The mice in normal saline group, as the blank control group, were injected with 250 μL normal saline. The mice in Taxol group were injected 250 μL of Taxol (PTX: 2 mg/kg) as the positive drug group. The different formulations were administrated intravenously by the tail vein every 2 days at a dose same as 2 mg/kg PTX. Measured the tumor volume and weight every 2 days using a Vernier caliper and an electronic scale. On the 13th day, 24 h after the last administration collected the blood samples, all the alive mice were sacrificed and the tumors and major organs were collected and weighed.

Relative tumor volume (RTV), Tumor growth inhibition (TGI) and the inhibition rate of tumor volume (IRTV) were significant indicators of antitumor efficacy *in vivo*, used following formula to calculate:

$$\mathrm{RTV}=\;\frac{TV_{i}}{TV_{1}}$$

$$\mathrm{TGI}\left(\%\right)=\left(\;1\;-\frac{T-T_{0}}{C-C_{0}}\;\right)\times \;100\%$$

IRTV (%)=( 1− TV aTVb )× 100%

Where *TV*_*1*_ and *TV*_*i*_ were the average tumor volume of the first day and 2, 4, 6, 8,10, 12 days, respectively. *TV*_*b*_ and *TV*_*a*_ were the change of the before and after treatment in mice tumor volume.Where *T*_*0*_ and *T* represented the treatment group before and the last day of the treatment of the average tumor volume, respectively. *C*_*0*_ and *C* on behalf of the blank control group before and after treatment the average tumor volume.

Additionally, the inhibition rate of body weight (IRBW) was calculated according to the following formula:

$$\mathrm{IRBW}\;\left(\%\right)=\left(\;1\;-\frac{BW_{a}}{BW_{b}}\;\right)\times \;100\%$$

Where *BW*_*b*_ and *BW*_*a*_ were the mice body weight before and after treatment, respectively.

All tumor tissue and major organ were fixed in 10% formalin solution, dehydrated, embedded in paraffin, cut into sections and stained with hematoxylin and eosin (H&E), and under a microscope (NIKON Eclipse Ci) to observe and photograph. Tumor sections were dewaxed, rehydrated, and incubated for immunofluorescence staining with TUNEL assay kit (Roche) and CD31 antibody. The nuclei were stained by the addition of DAPI by TUNEL assay.

### 2.11 Statistical Analysis

All data are expressed in terms of means ± standard deviation (SD). Statistical significance was assessed by the Student’s *t* test and one-way analysis of variance (ANOVA). *p* < 0.05 was considered statistically significant.

## 3 Results and Discussion

### 3.1 Characterization of Liposome

#### 3.1.1 Particle Size, Zeta Potential and Morphology

**FIGURE 1:**
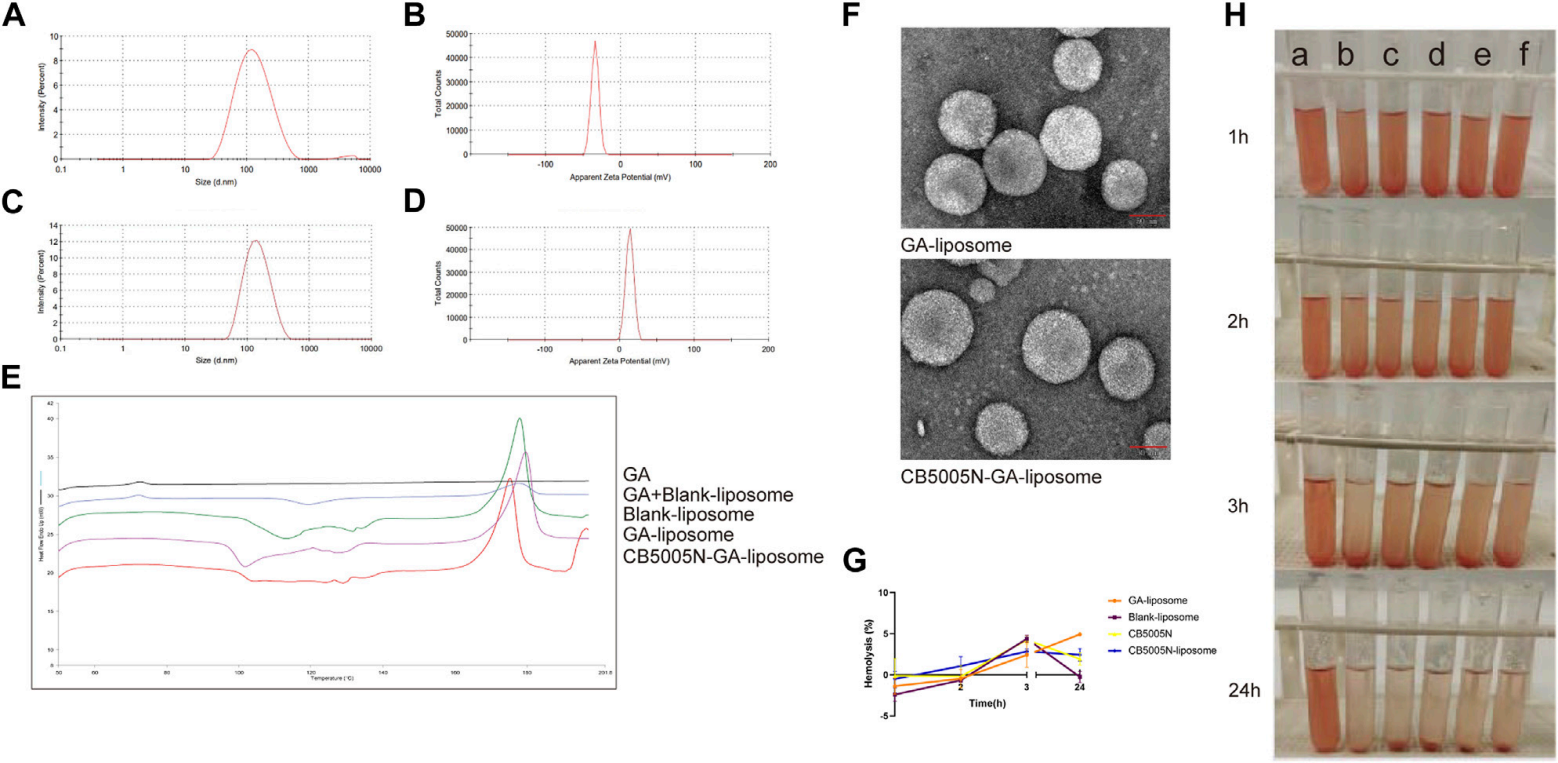
**(A–D)** Characteristics of GA-liposome and CB5005N-GA-liposome.**(E)** DSC of different preparations.**(F)** The TEM photos of GA-liposome and CB5005N-GA-liposome (scale bar = 50 nm). **(G)** Hemolysis (%). **(H)** Hemolysis assays (a: deionized water, b: saline, c: Blank-liposome, d: GA-liposome, e: CB5005N-liposome, f: CB5005N-GA-liposome).

The particle size of GA-liposome and CB5005N-GA-liposome were about 117–134 nm with the PDI less than 0.3, and the zeta potential was between −44 and 14 mV in Table 2. It is suggested that the size distribution of liposomes targeted breast cancer therapy is acceptable. The zeta potential and particle size distribution of GA-liposome and CB5005N-GA-liposome by DLS were displayed in Figure 1A−D. The morphological characteristics of liposomes were observed under TEM. In morphology, the liposomes were spherical in shape, well in dispersion and uniform in size distribution (Figure 1F).

**TABLE 2:** Mean particle size, PDI and zeta potential of Blank-liposome, GA-liposome and CB5005N-GA-liposome (Mean ± SD, *n* = 3).

Preparation	Size (nm)	PDI	ZP (mV)
Liposome	95.28 ± 0.85	0.14 ± 0.01	−19 ± 0.35
GA-liposome	117.43 ± 0.49	0.212 ± 0.012	−42.33 ± 1.63
CB5005N-GA-liposome	134.07 ± 1.4	0.21 ± 0.01	13.4 ± 0.4

The size and zeta potential of the nanocarriers are key factor in the biological processes such as biodistribution, blood circulation and tumor cellular uptake (Duan and Li, 2013; Nair et al., 2014). In this research, the average particle size of formulations was approximately 117–134 nm, within the acceptable range, besides, after modifying with CB5005N on the surface of GA-liposome, the size won’t change significantly. The study illuminated that the smaller the size of the nanoparticles, the more favorable for cell uptake. When the size of the nanoparticles is too small (<20 nm), the nanoparticles are more likely to be quickly cleared by mononuclear phagocytes (MPS), and they are easy to occur a large number of aggregations of liver, lungs and kidneys. When the particle size of nanoparticles is about 100 nm, it can show better long circulation effect and tumor targeting.

The main forces in the nanoparticles are the van der Waals force and the electrostatic repulsion force between the electric double layers, and these forces are related to the stability of colloidal dispersion. The strength of the mutual repulsion or attraction between particles is characterized by measuring the Zeta potential in the liposome system. The smaller the molecule or dispersed particle, the higher the absolute value of Zeta potential, and the more stable the nanoparticle system (Wu et al., 2018). Conversely, the lower the absolute value of Zeta potential, the more tend to flocculate or break between liposome particles, namely, the attraction exceeds the repulsive force. The absolute value of Zeta potential represents the stability of colloid system, and positive or negative represents the types of charge the particles carry. For the body, because the surface of the cell membrane is negatively charged, and the particles with cations on the surface have the ability to disrupt the cell microenvironment, and thus have cytotoxicity. As the delivery form of cationic liposomes, many researchers had designed antineoplastic drugs to increase the drug uptake of tumor cells and enhance their anti-tumor activity.

#### 3.1.2 Entrapment Efficiency

HPLC method was developed for the determination of the EE of liposomes for GA. The experimental results suggested that the modified GA-liposome and unmodified GA-liposome encapsulated the GA, the entrapment efficiency about 75%, remarkably increase the solubility of the GA. There was no remarkable difference in EE between unmodified and modified liposomes, it is suggested that the surface modification of polypeptide had no effect on EE of GA-liposome.

#### 3.1.3 Differential Scanning Calorimetry (DSC)

Differential scanning calorimetry (DSC) is commonly used to characterize nanoparticles. The application principle of DSC is that different lipids and crystal forms have different melting points and enthalpies, which are used to study the state and crystal form of lipid matrix in nanoparticles, as well as the existence form of coated substances in the matrix (Agrawal et al., 2010). The results of differential scanning calorimetry (DSC) were shown in Figures 1E GA showed an endothermic peak at 60°C–80°C, there were an endothermic peak of GA at 60°C–80°C and two endothermic peaks of blank liposome and freeze-drying protectant at about 120°C and 180°C of the physical mixture of GA and blank-liposome. Blank-liposome, GA-liposome and CB5005N-GA-liposome didn’t show the endothermic peak of GA. That is to say, no endothermic peak of GA was found in samples, indicating that GA was successfully encapsulated in liposomes without leakage. The results showed that GA was an amorphous state, which was encapsulated in liposomes.

#### 3.1.4 Hemolysis Assays

Hemolysis test is an important part of the safety evaluation of drugs before entering the market. In this experiment, the preparations were in direct contact with red blood cells to evaluate the biosafety of carrier materials. The effect of the preparation on the activities of red blood cells, coagulation factors and various enzymes in the blood was judged by calculating the degree of hemolysis. In the blood safety experiment, because the normal saline is an isotonic solution, the normal saline group was used as the negative control group without complete in hemolysis, and the deionized water was used as the positive control group with complete hemolysis. When the hemolysis rate was less than 5%, it showed that the blood safety of the preparation was well and would not produce hemolysis. In this experiment, the blood safety of the preparation was well, when there was no hemolysis, the absorbance of the preparations group was not much different from that in the normal saline group. The hemolysis test showed that the preparation had good biocompatibility and blood safety (Figures 1G, H).

### 3.2 Cell Uptake Study

Cou-6 is a derivative of phenylpyrrolidone coumarins, with excitation wavelength 360 nm and emission wavelength 477 nm. As a lipid-soluble laser dye, it has the advantages of high laser conversion and stable performance. It can be quantitatively determined by HPLC combined with ultraviolet detector or fluorescence detector. It has high sensitivity and widely used in the study of cell uptake (Khaled et al., 2016; Tian et al., 2016; Ding et al., 2017; Du et al., 2018). Chose high fluorescence intensity and low leakage rate green fluorescent markers Cou-6 used in the study of cellular uptake (Gao et al., 2013), and previously represented as the appropriate concentration (0.05 μg/ml) (Kebebe et al., 2019). Cell uptake was studied in human breast carcinoma MDA-MB-231 cells and mice breast cancer 4T1 cells. The two types of cells are triple negative breast cancer cells, have a higher risk of distant metastases, as well as the probability of visceral and brain metastasis was also higher (Bao et al., 2014), estrogen receptor (ER) and progesterone receptor (PR) and proto-oncogenes Her-2 immunohistochemical test results are negative (Miao et al., 2015), thus this result can also provide basis for the treatment of triple negative breast cancer.

**FIGURE 2:**
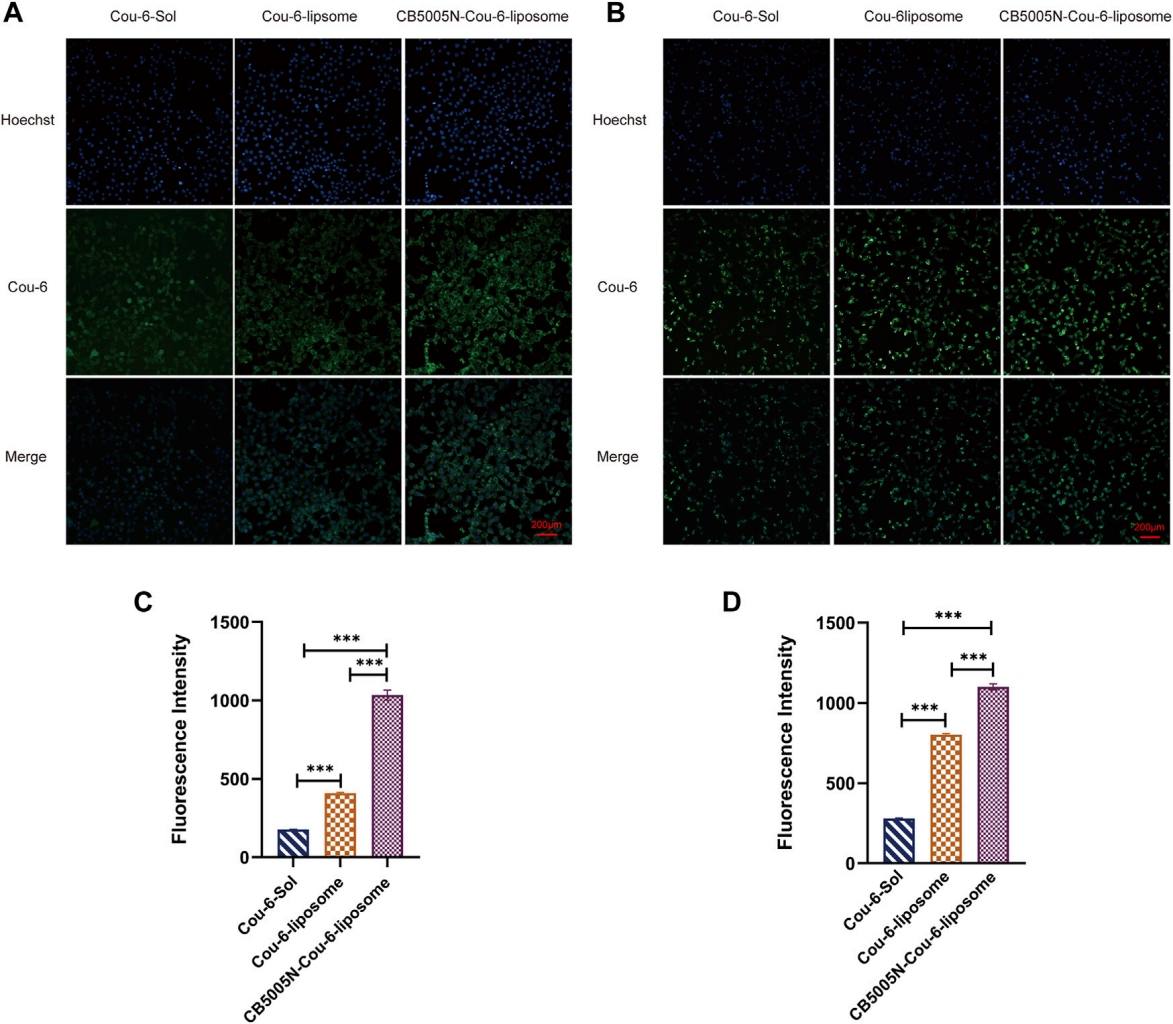
**(A)** The pictures of cell uptake by 4T1 cell after 4 h incubation. **(B)** The images of cell uptake by MDA-MB-231 cell after 4 h incubation. **(C)** Fluorescence values of Cou-6 in 4T1 cells in each group. **(D)** Fluorescence values of Cou-6 in MDA-MB-231 cells in each group. (Results are expressed as mean ± SD, *n* = 6. Scale bar = 200 μm) ***p < 0.001.

As shown in Figure 2, The same concentration of Cou-6 was taken in 4T1 and MDA-MB-231 cells, respectively. At all points in time, the intake of polypeptide decorated liposomes in 4T1 and MDA-MB-231 cells was obviously higher than that of solution and undecorated liposomes, that is to say, the green fluorescence intensity of CB5005N-Cou-6-liposome group was the strongest, followed by Cou-6-liposome group, and the weakest in Cou-6-Sol group. It is suggested that the internalization of the polypeptide modified liposomes had higher efficiency than other groups. This could account for the following reasons: cell by passive diffusion mechanism to absorb Cou-6-Sol, nevertheless, Cou-6-liposomes and CB5005N-Cou-6-liposomes were entered by nonspecific endocytosis into cells. The surface charge of liposome modified by CB5005N changed from negative to positive, the surface of the cell membrane carried a negative charge, thereby the nanoparticles with positive charge on the surface were easier to enter cells, thus increasing cell uptake.

### 3.3 Nuclear Targeting

**FIGURE 3:**
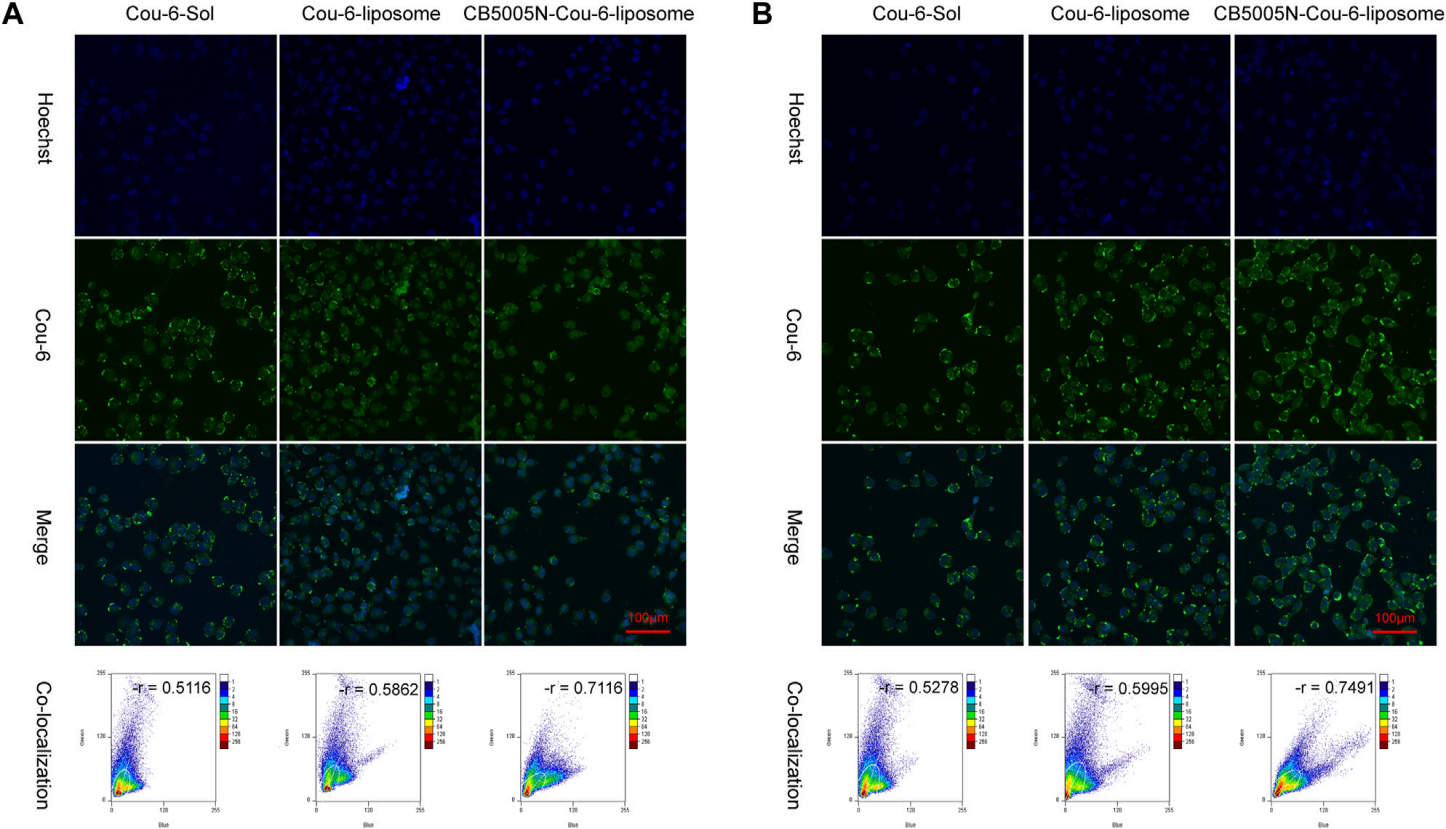
**(A)** The images of nuclear targeting by MDA-MB-231cell after 4 h incubation; **(B)** The images of nuclear targeting by MDA-MB-231 cells after 8 h incubation. (Scale bar = 100 μm).

The nuclear localization was studied by CLSM. Figure 3 showed images of various Cou-6 loaded liposomes. Nucleus were stained with Hoechst 33342. It is noteworthy that the green fluorescence intensity of CB5005N-Cou-6-liposome group was the strongest, followed by Cou-6-liposome group, and the weakest in Cou-6-Sol group. These results were in line with the cellular uptake (Figure 2), which indicated that CB5005N modified liposomes could effectively promote the accumulation of liposome in cells and nuclei. It is suggested that liposomes modified with polypeptide had higher efficiency of nuclear localization than Cou-6-Sol and Cou-6-liposome.

### 3.4 *In vitro* Evaluation of Cytotoxicity

Investigated the cytotoxicity of GA-Sol to MDA-MB-231 cells. Test results indicated that GA-Sol had a wide range of concentration on MDA-MB-231 cells and the 50% inhibitive concentration (IC50) was larger, while the concentration of GA was 0.2, 0.4, 0.6, 0.8 and 1.0 μg/ml, respectively. Therefore, the cytotoxicity of different preparation groups in these five concentration gradients was investigated in turn, and the IC50 values of different preparation groups were calculated. When investigated the cytotoxicity of GA-Sol to 4T1 cells, the initial concentration of GA was also set to 0.2, 0.4, 0.6, 0.8, 1.0 μg/ml. The results suggested that the survival ratio of 4T1 cell was lower than 50% when the concentration of GA was 0.4 μg/ml. Hence, the concentration range of GA was readjusted. That is to say, 4T1 cell was more sensitive to GA, and IC50 was narrow within the scope of 0.35–0.4 μg/ml. Under the condition of small IC50 value and narrow range, three concentrations (0.1, 0.35 and 0.6 μg/ml) were selected to further investigate the cytotoxicity of different preparations on 4T1.

**FIGURE 4:**
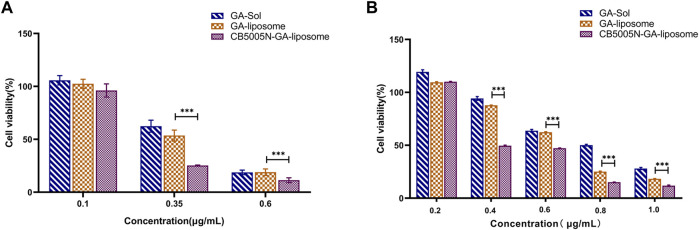
**(A)** Cell survival rate of 4T1 cells; **(B)** Cell survival ratio of MDA-MB-231 cells. (Results were expressed as mean ± SD, *n* = 6). ***p < 0.001.

*In vitro* antitumor activities of GA-Sol, GA-liposome and CB5005N-GA-liposome against 4T1 and MDA-MB-231 cells were determined by the above concentrations after 24 h incubation. As shown in Figure 4, GA for 4T1 and MDA-MB-231 cells cytotoxic effect also represented the concentration dependence. With the increase of concentration of GA, the cell survival ratio of 4T1 and MDA-MB-231 cells gradually decreased. Under the same concentration, compared with the solution group, both the GA-liposome group and the CB5005N-GA-liposome group effectively reduced the 4T1 and MDA-MB-231 cells viability, and with the increase of GA concentration, the inhibition was enhanced. The cytotoxicity of GA was significantly increased after GA was encapsulated into liposome, which could be contributed to the passive targeting of nanoparticles, thereby enhancing the antitumor activity. What’s more, liposome modified with CB5005N demonstrated a higher lethality on two types of cells in comparison to unmodified liposome, the results indicated that the surface of liposome modified with CB5005N enhanced the targeting efficiency and the intracellular concentration of GA. Therefore, these studies demonstrated that liposomes modified with CB5005N could increase the intake of two types of cells, thereby increasing intracellular GA concentration and ultimately leading to enhance cytotoxicity, which help to improve the treatment effect of tumor. In short, the cell toxicity experiments suggested that CB5005N modified liposome had a desired inhibitory effect on two types of breast cancer cells, and showed better antitumor activity *in vitro*.

### 3.5 *In-vivo* Imaging

Breast cancer model was established by subcutaneous injection of 4T1 cells into BALB/c nude mice. These mice are absent thymocytes and no hair all over the body, which has been widely used in the study of drug distribution and efficacy *in vivo* (Chang et al., 2015; Tanaka et al., 2017; Wang et al., 2019). In mice Breast cancer, the growth and metastasis characteristics of 4T1 cells in BALB/c mice are very similar to human breast cancer. Dir was used as a fluorescence probe for real-time imaging *in vivo*. Dir is a near infrared fluorescent dye with maximum fluorescence emission in the near infrared region. Compared with visible light, the light in the near infrared region is easier to penetrate many tissues of the body, making it easier to detect tagged objects in the body (Liu and Wu, 2016). And the near infrared excitation and emission wavelengths of Dir can effectively reduce the interference of autofluorescence in animals (Sharma et al., 2006).

**FIGURE 5:**
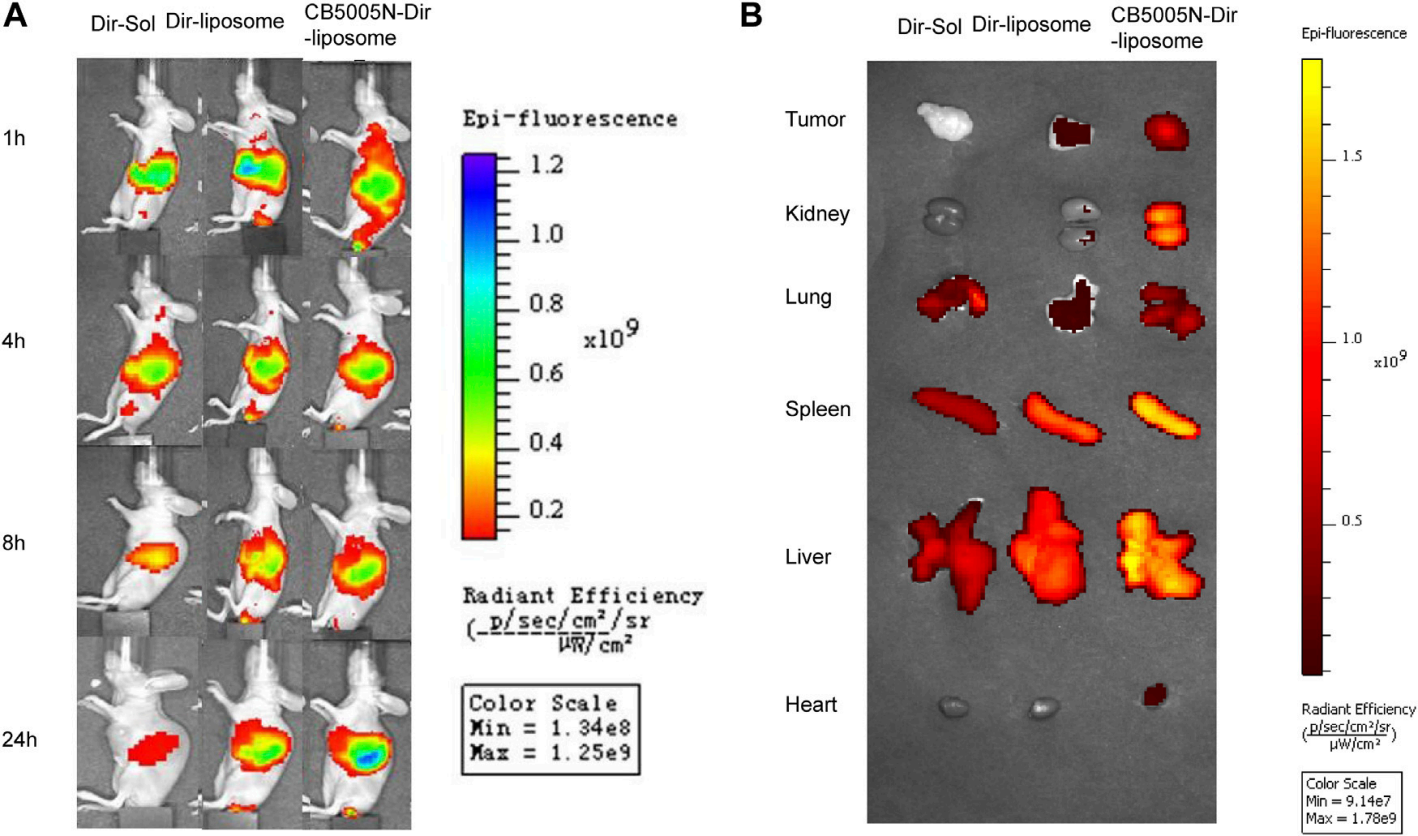
**(A)** Comparison of tumor fluorescence intensity in tumor bearing mice before and after drug administration; **(B)** The pictures of the tumors and organs removed from mice after 24 h (*n* = 3).

The biological distribution of Dir-Sol, Dir-liposome and CB5005N-Dir-liposome groups was observed with IVIS animal *in vivo* imager at different time points of 1, 4, 8, and 12 h, and then compared the targeting abilities of the modified group with unmodified group *in vivo*. A color scale is used to measure the intensity of fluorescence, the change from red to blue represents the gradually increasing intensity of fluorescence. As shown in Figure 5A. In the Dir-Sol group, there was no fluorescence at the tumor site, indicating that the drug solution had no tumor-targeting property, and the fluorescence intensity of the nude mice significantly decreased after 4 h, indicating that Dir solution was quickly cleared in the body. In Dir-liposome group, as time went on, there was a small amount of fluorescence at the tumor site from 4 to 24 h, suggesting that the drug could be delivered to the tumor site by small amount of liposome. When all the liposome groups were compared with the solution group, the Dir in the solution group was cleared rapidly after 8 h, and the fluorescence intensity was significantly weaker than that in the liposome group, while the liposome group still had a strong fluorescence intensity at 24 h, indicating that liposome can achieve the effect of long circulation *in vivo*, reduce the clearance rate of the drug in the body, and prolong the retention time in the body. However, as can be seen from the picture (Figure 5B), in addition, the fluorescence intensity was obvious not only in the tumor site, but also in the major organs of nude mice, indicating that the drug has a certain accumulation in the major organs.

### 3.6 *In vivo* Anti-Tumor Effect

**FIGURE 6:**
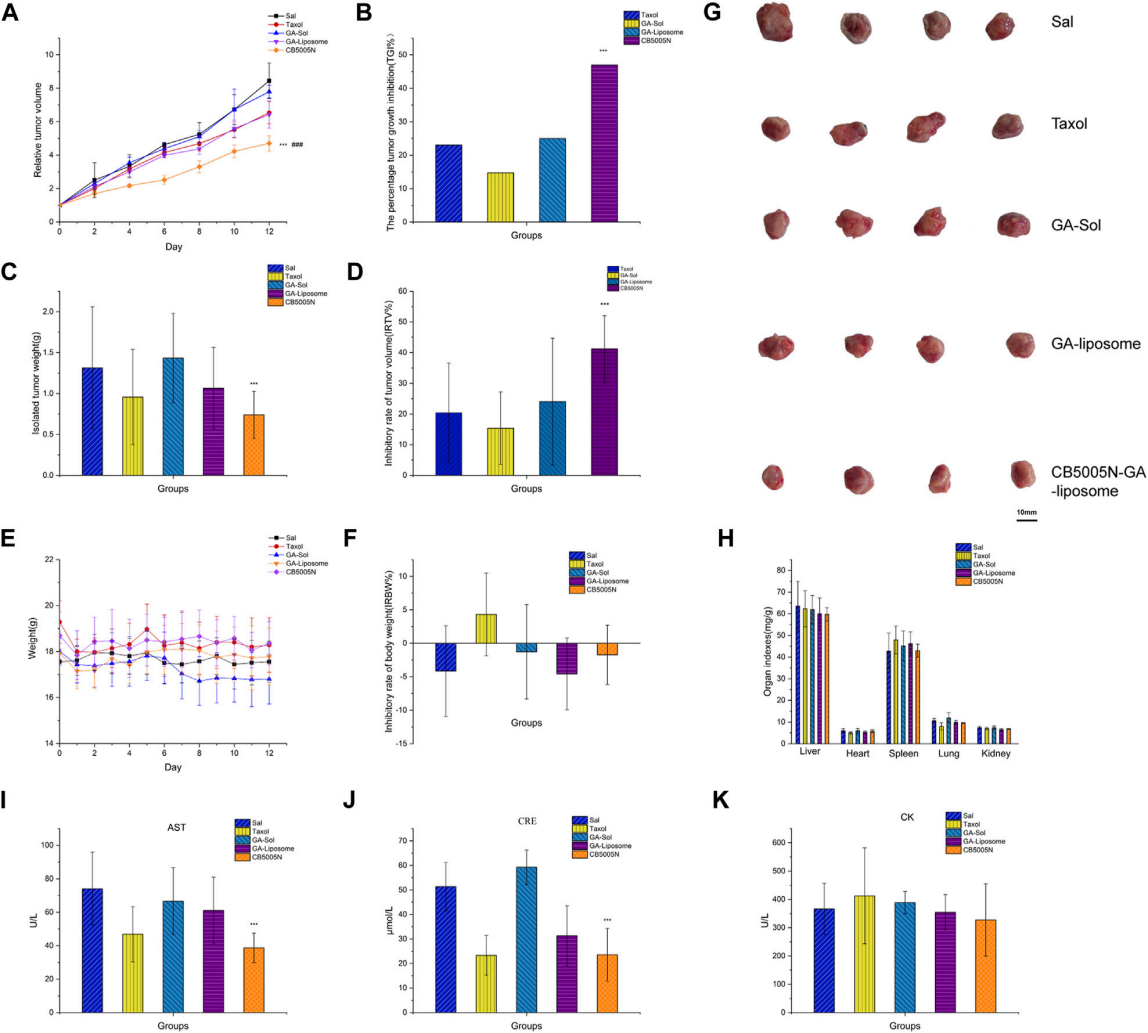
Antitumor effects *in vivo*. (**(A)** Relative tumor volume **(B)** The percentage tumor growth inhibition **(C)** Isolated tumor weight **(D)** Inhibitory rate of tumor volume **(E)** Weight **(F)** Inhibitory rate of body weight **(G)** Isolated tumor (H) The viscera index of mice **(I–K)** The biochemical markers). Results were expressed as mean ± SD, *n* = 4 (***p < 0.001 vs GA-liposome, ###*p* < 0.001 vs Taxol).

*In vivo* anti-tumor efficacy of GA-liposomes and CB5005N-GA-liposomes was assessed in a mouse model, and saline group was the blank control, Taxol group was the positive drug group. CB5005N-GA-liposome has high targeting efficiency and was supposed to achieve better treatment effect and lower adverse reactions. The test results were manifested in Figure 6A, the tumor growth of blank control group was rapid, while the tumor growth of other treatment groups was inhibited to varying degrees. There was little effect on tumor size among groups during the first 3 days, however, at the 13th day, tumor growth was significantly reduced in targeting decorated group. Further down, according to ethical requirements (maximum tumor volume less than 1,000 mm^3^), the mice were euthanized on the 13th day. We also found that tumor size in the GA-liposome group was outstandingly reduced in comparison with that in the GA-Sol group, and those in CB5005N-GA-liposome treatment group were hugely reduced compared with the GA-Sol treatment group. The tumor growth of liposome groups was slower than that of solution group, and the tumor volume of CB5005N-GA-liposome group was obviously smaller than that of GA-liposome group (*p* < 0.05). These results indicated that the antitumor activity of GA was enhanced by nanocarriers, and the active targeting mechanism may be an important reason for enhancing the antitumor activity of CB5005N-GA-liposome.

The percentage tumor growth inhibition (TGI%) by Taxol, GA-Sol, GA-liposome, and CB5005N-GA-liposome were 23.06, 14.72, 24.99 and 46.97%, respectively (Figure 6B). Compared with Taxol, solution and non-targeted liposomes, targeted liposomes had stronger inhibition on tumor growth and exhibited higher antitumor activity. As shown in Figures 6C, G, the mice treated with CB5005N-GA-liposome obtained relatively smaller tumor weight and size compared to other dosage forms, and the inhibition rate of tumor volume (IRTV%) by Taxol, GA-Sol, GA-liposome, and CB5005N-GA-liposome were 27.17, 3.62, 18.8 and 43.7%, respectively (Figure 6D). Compared with GA-liposome, CB5005N-GA-liposome had significant antitumor effect. The results concluded that liposomes can improve the inhibition ratio of GA, which was paralleled with the results of above.

One of the commonly used safety indicators is tumor-burdened mice body weight changes with time. The weight inhibition rate was calculated. During therapy period, GA-liposomes and CB5005N-GA-liposomes groups were observed no notable change in body weight (Figure 6E). Meanwhile, the body weight of the Taxol group showed decreased with IRBW of 5.61% (Figure 6F). The weight loss more of Taxol were likely due to the toxicity of solvent system. The liposomes groups were well tolerated, suggesting that the potential of target uptake of liposomes by normal cells may not cause a detectable damage. At the same time, drugs were embedded in liposomes, can significantly decrease the toxic and side effects of drugs. These results indicated that CB5005N modified GA-liposome delivery system was more effective and safer than Taxol. In order to detect the potential toxicity of different treatment groups mice, the histological analysis of main organs.

The viscera index of mice was calculated. In liver index, heart index, spleen index, the index of lung and kidney index, the saline and the targeted liposomes groups had no significant difference; while the Taxol group showed splenomegaly and inflammation in the lungs (Figure 6H). The serum CRE, AST, and CK levels were determined to further assess the potential toxicity of different preparations (Xiong et al., 2016). The serum CRE, AST, and CK were obtained from Nanjing Jiancheng Bioengineering Institute (China) and detected using multifunctional enzyme labeling instrument and ultraviolet spectrophotometer, all tests were carried out in accordance with the regulations of the manufacturers.

The serum CRE level in the saline group was higher than the liposomes groups, and the CB5005N-GA-liposome group significantly decreased the serum CRE activities compare to the GA-liposome group, probably due to the saline group was no-carriers, CB5005N-GA-liposome group was modified targeted peptide, leading to excretory function of the kidney is decreased. (Figure 6I). The serum AST activities were reduced in CB5005N-GA-liposome group (Figure 6J), probably owing to a hepatoprotective effect (Verçosa Junior et al., 2016). In addition, for CK, liposome group compared with control group (blank and positive control group) had no notable decrease (Figure 6K). Accordingly, CB5005N-GA-liposomes could obviously improve GA efficacy, and decrease its toxic and side effects of the inhibition of breast cancer.

**FIGURE 7:**
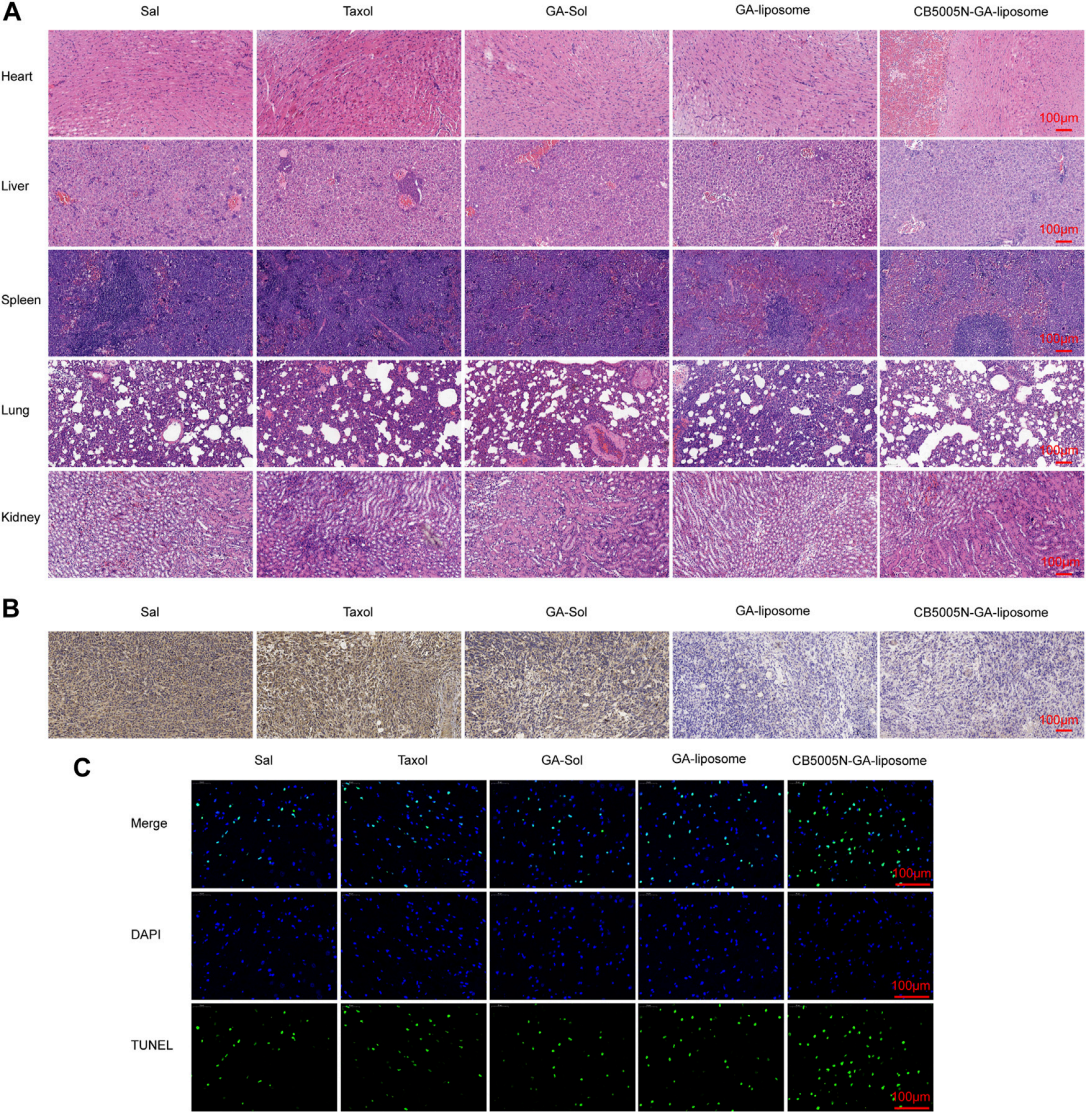
**(A)** H&E staining of major organs **(B)** After different treatments, tumor sections were stained with CD31 and **(C)** TUNEL, Scale bar = 100 μm. (*n* = 4).

In addition, H&E results were in line with the results (Figure 7A). Hematoxylin was acquired from Wuhan Biotechnology Co., Ltd. (China). The main organs of the modified liposome group with CB5005N showed no obvious histological changes, suggesting that targeted liposome did not produce significant systemic toxicity.

### 3.7 Immunohistochemistry Analysis

Immunohistochemistry analysis by measuring and CD31 staining, which was obtained from Abcam (Cambridge, UK). CD31, as the most sensitive and specific endothelial marker, can evaluate angiogenesis. Hence, in order to confirm whether the anti-tumor effects of different formulations associated to the anti-angiogenesis effect, the blood vessels of 4T1 tumor were stained by using the anti-CD31 antibody. The results indicated that both GA-liposome and CB5005N-GA-liposome group, the tumor blood vessels were affected by different degree. Consistent with previous reports, GA reduced vascular density in CD31-related immune-histological studies (Lu et al., 2013). It can be seen from Figure 7B, CB5005N significantly enhanced GA-liposome antiangiogenic effect.

Generally speaking, few TUNEL positives were showed in the saline group. On the contrary, the GA treatment group showed the TUNEL positive (Figure 7C). The images also showed that liposome loaded GA caused more apoptosis of cancer cells than the same dose of solution and Taxol treatment. It^’^s noteworthy that the tumor tissues of CB5005N-GA-liposome group exhibited the most apoptosis cells in mice compared to the other groups. The results of the cell apoptosis trends were in line with the results of previous *in vivo* antitumor.

## 4 Conclusion

In a nutshell, we prepared an original CB5005N decorated liposome delivery system to enhance the therapeutic effects of GA on breast cancer. The prepared CB5005N-GA-liposome had high encapsulation efficiency, narrow particle size distribution, great biocompatibility, high cytotoxic effect *in vitro*. Cellular uptake results demonstrated that liposome modified by CB5005N enhanced intracellular accumulation. The nuclear targeting experiments proved CB5005N-GA-liposome had significantly targeting function. In mouse 4T1 tumor model, it had strong anti-tumor effect and good targeting to tumor tissues and cells. The above results demonstrate that CB5005N-GA-liposome might be a novel anti-tumor preparation for breast cancer with high prospect of clinical application.

## Data Availability

The original contributions presented in the study are included in the article/Supplementary Material, further inquiries can be directed to the corresponding author.
